# High-energy density cellulose nanofibre supercapacitors enabled by pseudo-solid water molecules

**DOI:** 10.1038/s41598-024-61128-w

**Published:** 2024-05-06

**Authors:** Mikio Fukuhara, Tomonori Yokotsuka, Masahiro Morita, Tatsunori Ito, Minoru Yada, Takeshi Nakatani, Toshiyuki Hashida

**Affiliations:** 1 New Industry Creation Hatchery Center, Tohoku University, Aoba, Sendai 980-8579 Japan; 2 Fuji Innovative Materials Research Laboratory, Nippon Paper Industries, Co. Ltd., Fuji, 417-8520 Japan

**Keywords:** Nanofibre supercapacitor, Pseudo-solid water molecule, Energy density, Renewable electronic device, Biotechnology, Energy science and technology, Materials science, Nanoscience and technology

## Abstract

Compared with conventional electrochemical supercapacitors and lithium-ion batteries, the novel amorphous cellulose nanofibre (ACF) supercapacitor demonstrates superior electric storage capacity with a high-power density, owing to its fast-charging capability and high-voltage performance. This study unveils introduces an ACF supercapacitor characterised by a substantial energy density. This is achieved by integrating a singular layer of pseudo-solid water molecules (electrical resistivity of 1.11 × 10^8^ Ω cm) with cellulose nanofibers (CNFs), establishing forming an electric double layer at the electrode interface. The enhanced energy storage in these high-energy density capacitors (8.55 J/m^2^) is explicated through the polarisation of protons and lone pair electrons on oxygen atoms during water electrolysis, commencing at 1.23 V. Improvements in energy density are attainable through CNF density enhancements and charging-current optimisation. The proposed ACF supercapacitor offers substantial promise for integration into the power sources of flexible and renewable paper-based electronic devices.

## Introduction

Batteries are devices that convert the chemical energy of materials into direct current through chemical reactions. This transformation predominantly occurs in primary batteries. In contrast, secondary batteries have the capability to convert electrical energy back into chemical energy for storage purpose^1–3^. Some common types include lead acid^4^, lithium-ion rechargeable^5^, nickel hydrogen^6^, sodium sulfur^7^, and solid-state batteries^8^. Recent advancements have resulted in the development of aqueous lithium ion—batteries with hydrate-melt electrolytes, which exhibit high energy densities (> 130 Wh/kg) and voltages (~ 2.3‒3.1 V)^9^. However, supercapacitors are promising candidates for a new generation of energy storage devices due to their superior power density, stability, longevity, and eco-friendliness. Despite these advantages, it is important to note that their energy density is 1‒2 orders of magnitude lower than that of lithium-ion batteries. The object is to develop a renewable electricity storage device that overcomes the constraints of existing systems. Unlike conventional chemical batteries and supercapacitors, this study is dedicated to the advancement on the development of solid-state physical supercapacitors, which are capable of fast charging up to 500 V. These devices are constructed using amorphous blackish alumina^10,11^ and transparent cellulose nanofibres (CNFs)^12,13^. They leverage the unique nanostructured surfaces and high work functions of these materials to efficiently create electric double-layer capacitors (EDLCs). The quantum-size and enhanced electro-absorption effects of these materials facilitate the storage of substantial amounts of electricity, with recorded values of 1710.3 and 1416.7 mJ/m^2^, respectively^13,14^. Recently, Teng et al.^15^ grew MnO_2_ nanowires as positive electrodes and polyaniline nanoparticles as negative electrodes on a three-dimensional carbonised wood matrix, and formed numerous electrochemical exchange reactive sites on the porous wall in contact with the aqueous electrolyte. This resulted in a high-areal capacitance of 1,721 mF/cm^2^ at 2 V. Li et al.^16^ also reported that methyl cellulose-based, quasi-solid-state supercapacitors with nitrogen and boron dual-doped carbon electrodes exhibited a high energy density of 572 F/g at 0.5 A/g. This study addresses the limitations of supercapacitors, by aiming to: 1) develop a CNF-based supercapacitor that utilizes only the polarizability of bound water, thereby avoiding conventional ionic electrolyte solutions; and 2) investigate the role of pseudo-solid water molecules in enhancing the performance of the supercapacitor. Recent studies, such as those by Liu et al.^17^, have demonstrated that thin films of nanometre-scale protein wires can produce continuous electric power, highlighting the potential of microbe-derived materials in energy generation. Additionally, research on materials confined within carbon nanotubes has revealed a blurring of the solid‒liquid phase distinction under certain conditions^18,19^. Furthermore, investigations by Harrellson et al.^20^ highlighted the multifaceted role of water in biological materials, suggesting that hydration forces can lead to the formation of a “hydration solid” with unique properties.

## Results and discussion

### I–V characteristics for dried and moist specimens

**Figure 1:**
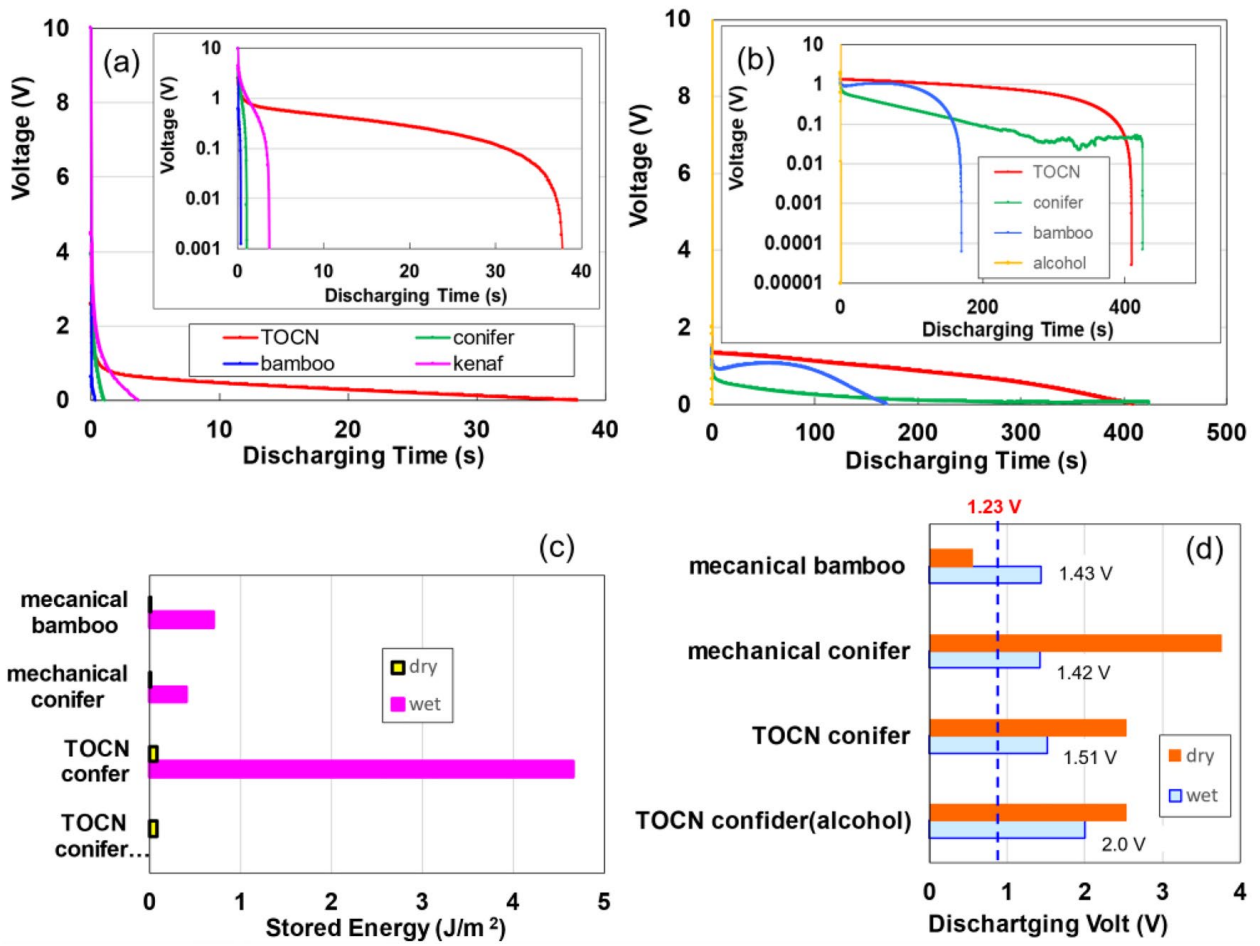
(**a**) Discharge behaviours of dry TOCN, mechanically defibrated kenaf, conifer, and bamboo at a constant current of 1 µA following charging for 50 s at 2 mA- and 10 V. (**b**) Discharge behavior of wet TOCN, mechanically defibrated conifer and bamboo, and alcohol-treated TOCN at a constant current of 1 µA after charging for 50 s at 24 mA- and 10 V. (**c**) Comparative analysis of energy storage in four dry versus three water-treated and alcohol-treated specimens. (**d**) Comparative analysis of discharge voltage post *IR* drop for four dry versus three water-treated and alcohol-treated specimens.

Figure 1a shows the discharge characteristics of kenaf, conifer, bamboo, and 2,2,6,6-tetramethylpiperidine-1-oxyl radical (TEMPO)-oxidised cellulose nanofiber (TOCN) specimens under a constant current of 1 μA following charging for 50 s at 2 mA and 10 V. The TOCN specimen, composed of chemically disintegrated fibres, exhibits the highest discharge or storage capacity, surpassing those of the mechanically defibrated kenaf, conifer, and bamboo. Previous studies have elucidated the superior energy storage capabilities of TOCN^12,13^. The discharge behaviours of the conifer, bamboo, and TOCN specimens, after exposure to pure water and anhydrous alcohol vapours were evaluated under a constant current of 1 μA following charging for 50 s at 24 mA and 10 V, as shown in Fig. 1b. The discharge volume increased in the order of TOCN, conifer, and bamboo, with the TOCN specimen moistened with anhydrous alcohol exhibiting no discharge characteristics, highlighting water’s critical role in the functionality of TOCN supercapacitors. Figure 1c demonstrates the variance of charge amounts between dried and moistened specimens.

The stored energy of moist specimens increases by approximately 20­400 times compared with that of dried specimens, indicating that even a minor presence of water significantly enhances energy storage capabilities. The significantly electrical storage observed in the TOCN sample relative to the mechanically defibrated conifer and bamboo samples may be attributed to the fibre diameter of TOCN, which is approximately 3 nm^12^. This diameter is significantly different from those of conifer and bamboo, whose fibre diameters are in the range 20–60 nm. This discrepancy may potentially lead to a substantially reduced specific surface area for the conifers and bamboo fibres for TOCN one (see pore size distribution analysis in Supplementary Information [hereafter, referred to as (SI) Fig. S1]. Figure 1d illustrates the discharging voltage following an *IR* drop in the moist CNF specimens. The *IR* drop stems from internal charging in unsaturated cells as well as from the EDLC effect^21^. The initial voltage for discharging ranged from approximately 1.42–1.51 V.

### Effects of charging voltage and current for moist specimens

**Figure 2:**
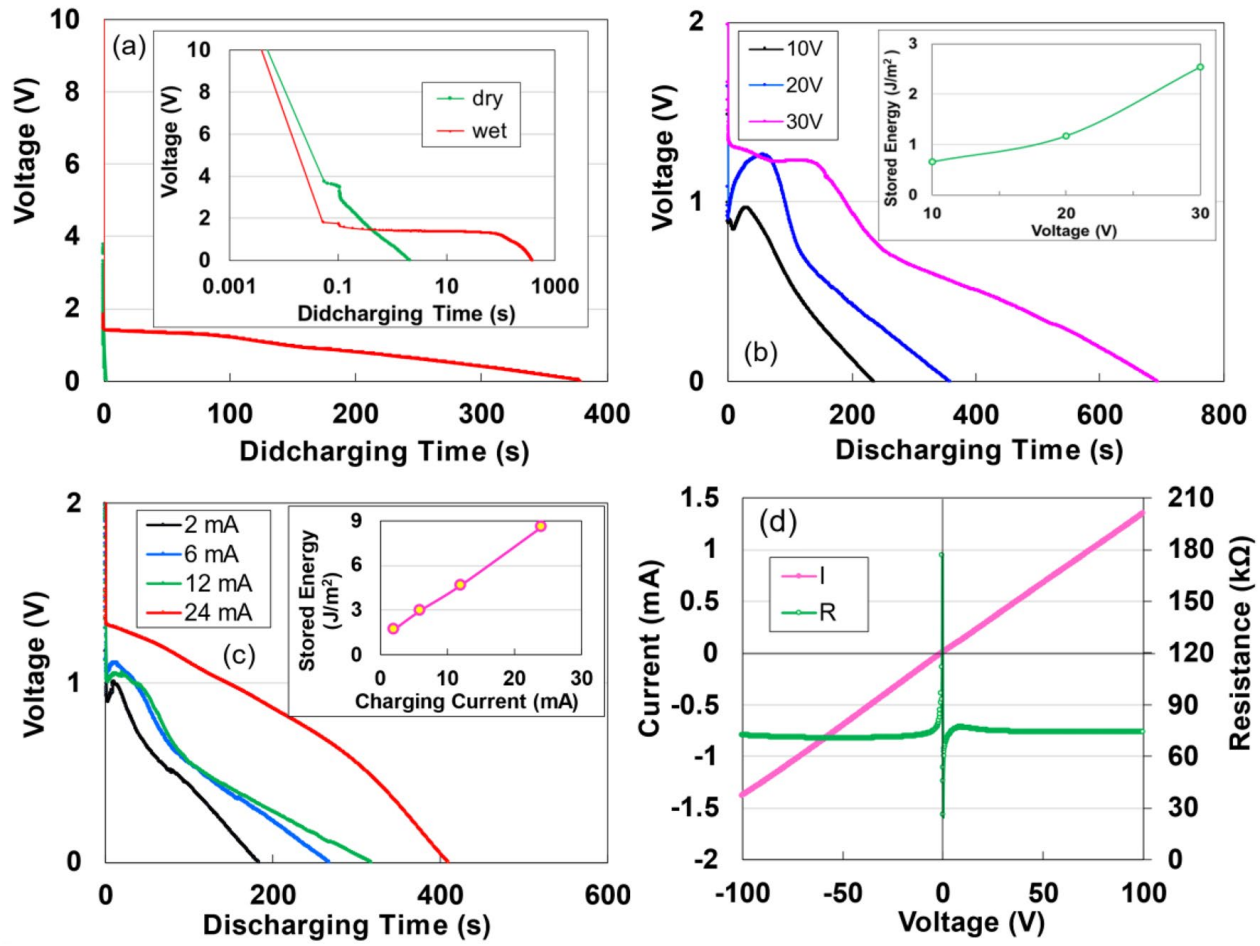
(**a**) Discharge profiles for dry and moist TOCN specimens subjected to a constant current of 1 µA after charging for 50 s charge at 2 mA- and 10 V. (**b**) Discharge profiles for moist TOCN specimens under a constant current of 1 µA following charging for 50 s at 24 mA across 10, 20, and 30 V. Inset: Correlation between the stored energy and charging voltage. (**c**) Discharge profiles for moist TOCN specimens at a constant current of 1 µA after charging for 50 s at 2, 6, 12, and 24 mA. Inset: Correlation between the stored energy and charging current. (**d**) *I*–*V* and *R*–*V* characteristics across a voltage range from − 100 to + 100 V.

Figure 2a illustrates the discharge characteristics of both dried and moist specimens using TOCN, which exhibited the highest charge storage capacity. The discharge curve of the dried specimen decreases parabolically over time, whereas the moist specimen maintains a discharge from 1.32 V for an extended duration, leading to enhanced energy density. The temporary increase in discharge voltage at any given voltage may be attributed to the averaging effect across numerous small capacitors. Figure 2b displays comparative data on the impact of charging voltages up to 30 V, under which no leakage between electrodes was observed at a constant current of 24 mA. The inset of Fig. 2b illustrates the dependency of the charging voltage on the final stored energy, indicating an increase as a function of voltage. Figure 2c demonstrates the relationship between discharging time and charging current; the inset shows that the stored electricity improves as a function of currents. The energy and power densities, obtained at 24 mA and 10 V (applied for 50 s) are 8.55 J/m^2^ and 4.43 × 10^−3^ W/m^2^, respectively. It is anticipated that the energy density will increase further with the augmentation of the charging current and the density of the TOCN sample. Figure 2d outlines the double* I*–*V* and *R*–*V* characteristics across a range from − 100 and + 100 V in air. The observed curves are asymmetric relative to zero bias, mirroring the Coulomb blockade behaviour^10^, similar to both the Coulomb blockade phenomena and the behaviour of dried TOCN supercapacitors^12^. This asymmetry may be related to the specimen’s size^22^. From the *R*–*V* curve, the electrical resistivity at + 100 V is identified to be 1.11 × 10^8^ Ω cm, closely approximating the value (1.52 × 10^8^ Ω cm^23^) of ice at 262 K.

### Complex evaluation of electric storage

**Figure 3:**
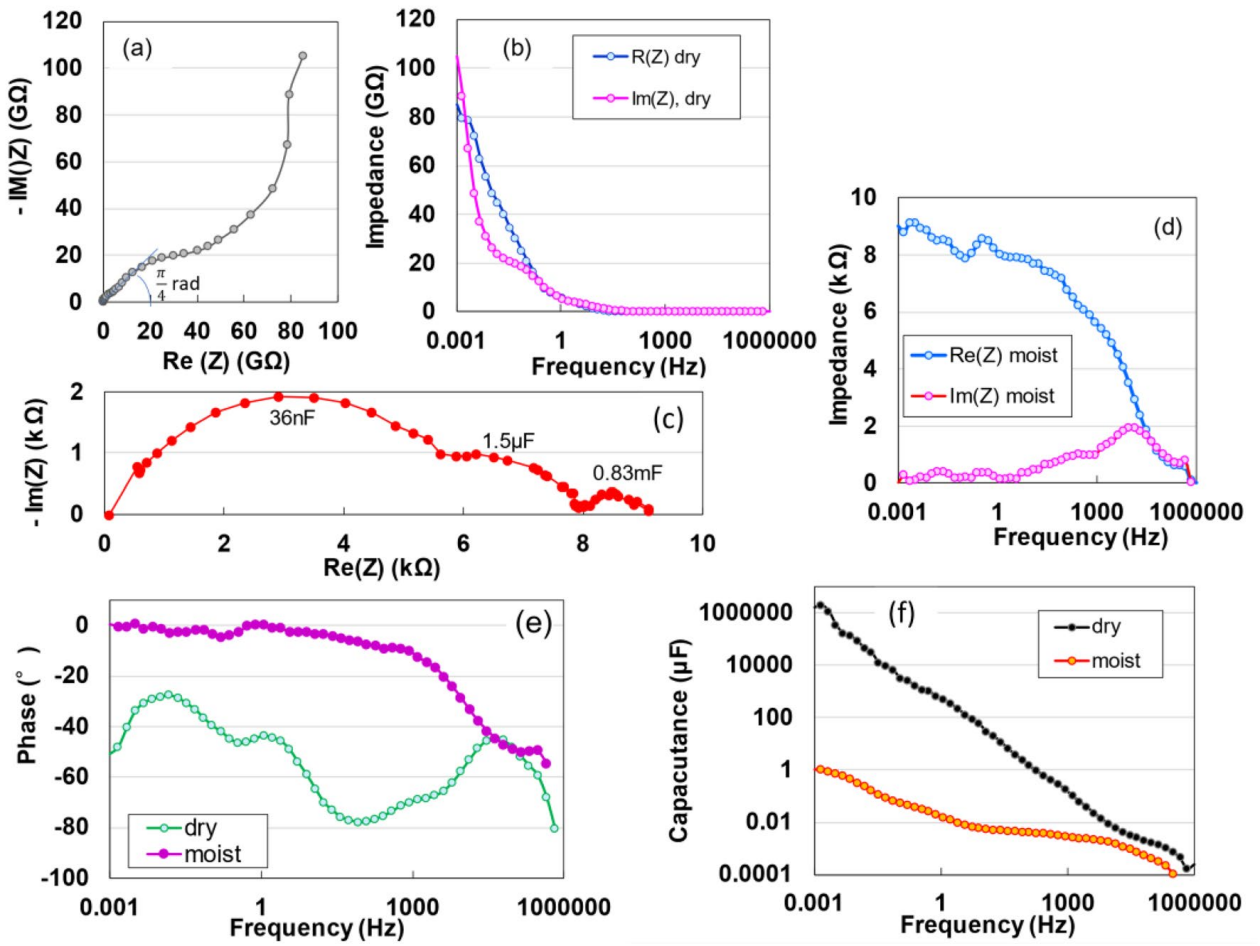
Electrostatic analysis of the TOCN specimen without destruction. Nyquist plots for dry (**a**) and moist (**c**) TOCN devices. Real and imaginary impedances for dry (**b**) and moist (**d**) TOCN devices, respectively. Frequency-dependent phase angle (**e**) and series capacitance (**f**) for both dry and moist TOCN devices.

To analyse the electrostatic contributions of dried and moist specimens, non-destructively, alternating current (AC) impedance measurements were conducted across a frequency range from 1 mHz to 1 MHz. Nyquist diagrams, influenced by various scan speeds and current densities, were generated. However, in this study, measurements were performed using a direct current (DC) supply of 10 V and a charging current of 10 mA. The impedance spectrum of the dried TOCN specimen (Fig. 3a) displays a distinctive pattern comprising a straight line with a π/4 rad slope, a semicircle, and a near-vertical line. This pattern is indicative of a series RC circuit configuration^10–12,24^. In contrast, the impedance spectrum of the moist specimen (Fig. 3c) features three distinct semicircles of varying sizes, representing large, medium and small contributions. The detailed electrostatic evaluation is described at Table S1 in SI. The Bode plot for the dried specimen (Fig. 3b) reveals a sharp increase in both the real and imaginary impedance at low frequencies. Conversely, the moist specimen (Fig. 3d) exhibits a gradual increase in real impedance starting from approximately 2 kHz, accompanied by a single peak in dielectric dispersion at 13.6 kHz within the imaginary impedance spectrum, a phenomenon attributed to interfacial polarisation. A notable decline in phase angle to − 90° with decreasing frequency observed in the dried specimen (Fig. 3e) provides additional DC charging evidence. This observation implies that capacitors within the dried TOCN specimen are arranged in a series circuit, where the total capacitance *C* is the sum of the capacitances of individual capacitors, that is $C = \sum\nolimits_{k = 1}^{n} {\,C} k = nC$^25^. Conversely, the capacitive behaviour within the frequency range starting from 1 Hz in the moist specimen, characterised by a phase angle that was almost 0°, suggests the presence of a parallel *RC* circuit. In the experimental curve shown in Fig. 3f, the parallel capacitance (C_s_) for the dried specimen exhibits a parabolic increase as the frequency decreases, attaining a value of 1.9 F at 1 mHz. In contrast, the parallel capacitance of the moist specimen achieves a maximum of only 1 μF, illustrating significant differences in the capacitive behaviour between dried and moist specimens. Furthermore, an information on the coulombic efficiency of devices is described at Table S2 in SI.

### Optimised structure of moist TOCN and its electric role

**Figure 4:**
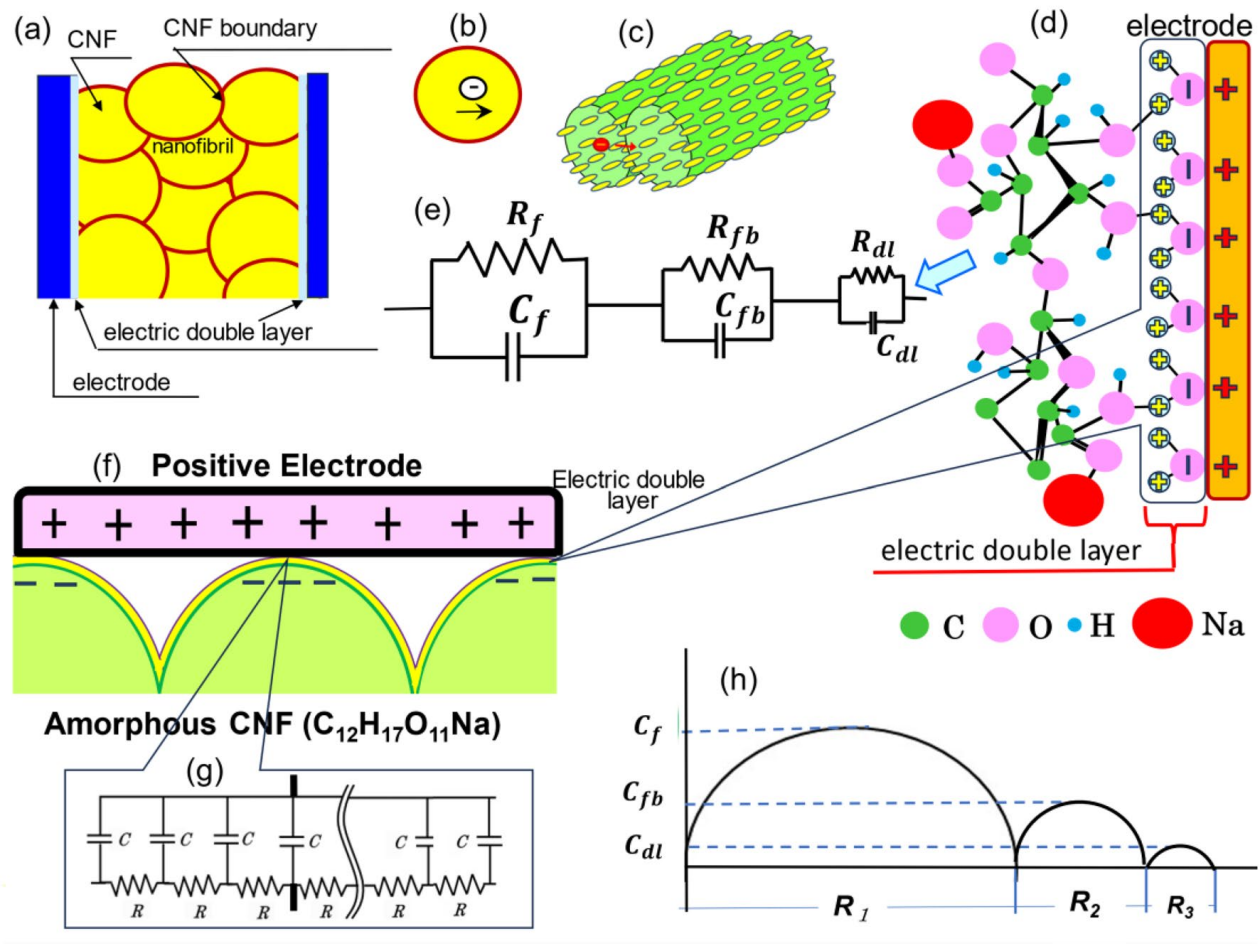
Schematic of electron conduction in a moist CNF supercapacitor. (**a**) Structural model of the supercapacitor. (**e**) Electrical equivalent circuit comprising series connections of three parallel circuits (nanofibril aggregate, CNF boundary, and electrode–electric double layer interface), each including resistors (R_f_ for nanofibrils (**b**), R_fb_ for the nanofibril boundary (**c**), and R_dl_ for the electric double layer (**d**)) and capacitors (*C*_*f*_ for nanofibrils, *C*_*fb*_ for the nanofibril boundary, and *C*_*dl*_ for the electric double layer). (**f**) Illustration of the microscopic electric energy storage approach adopted in this study. (**g**) Electric distributed-constant circuit of the moist CNF. (**h**) Diagram corresponding to the Nyquist plot in Fig. 3c.

In this study, the moist TOCN supercapacitor exhibited a considerable energy density, approximately 8.55 J/m^2^, though its power density was relatively lower. This discrepancy necessitates an exploration of the underlying reasons. The Nyquist diagram for the moist TOCN specimen, which features three semicircles as depicted in Fig. 3c, suggests a complex internal structure. This structure is deduced from Fig. 4a and is theoretically represented by an electrical equivalent circuit. The circuit is conceptualised as a series arrangement of three parallel circuits, each representing different components of the supercapacitor: the nanofibril aggregate, the CNF boundary, and the interface between the electrode and the electric double layer. Each parallel circuit comprises a resistor and capacitor, denoted as R_f_ and C_f_ for the nanofibrils, R_fb_ and C_fb_ for the CNF boundary, and R_dl_ and C_dl_ for the electrode interface, as illustrated in Fig. 4e. Understanding the electrochemical behaviour and storage capabilities of the supercapacitor hinges on the resistances and capacitances of these components R_f_ (Fig. 4b) and C_f_ for the nanofibrils, R_fb_ (Fig. 4c) and C_fb_ for the CNF boundary, and R_dl_ (Fig. 4d) and C_dl_ for the electrode interface. Figure 4h schematically depicts the microscopic electric energy storage mechanism, correlating with the findings from the Nyquist diagram. Through this detailed examination, we gain insights into the electrical and structural dynamics within the moist TOCN specimen, elucidating the factors contributing to the supercapacitor’s high energy density, despite its comparatively lower power density.

This study highlights the electric polarization of water molecules interacting with CNF molecules at the electrode interface in moist TOCN specimens. The oxygen atoms in the CNFs’ hydroxyl groups are equatorially arranged in the chair conformation of the glucopyranose rings. These oxygen atoms form robust hydrogen bonds with the hydrogen atoms of water molecules upon the introduction of a slight amount of water into the interface between the TOCN and its electrode. Notably, Mashl et al.^26^ reported that the water within carbon nanotubes (diameters < 0.86 nm) spontaneously crystallises, transitioning into a semiconductor phase. In a subsequent paper, this phenomenon will be described; attention will be given to quantify the *I*–*V* characteristics by applying direct currents in pure water confined into an extremely thinner cell.

In ice, the oxygen positions within the water molecule crystal structure are fixed, whereas the proton positions are disordered and not fixed. As a result, polarisation occurs owing to the orientation of protons in response to an applied electric field^27^. By analogy, it is inferred that the hydrated water at the electrode interface transitions into a pseudo-solid phase in the narrow confinement of 0.311 nm^28^ (discussed in the SI S7), such as that of an electrolyte, The lone paired electron of oxygen^29^ and the protons from hydrogen atoms in the pseudo-solid water molecules create an electric double layer at the electrode interface with positive charges, as shown in Fig. 4d. Ultra-high pure water with an electrical resistance above 18.2 MΩ·cm, is known to generate static electricity owing to polarisation, with H_2_O dissociating into 2H^+^ and O_2_^−1^^30^. Gouveia and Galembeck observed that the overall sample potential of hydrophilic particles is always negative, which may be attributed to the partitioning of OH^−^ and H^+^^31^. This suggests the possibility of electrical polarisation in pseudo-solid water molecules using the electrical energy charged by the TOCN supercapacitor. As observed in Fig. 1d, the initial discharge voltages of the moist TOCN supercapacitor after a 10 V charge fall to values in the range of 1.43–1.51 V, closely aligning with the standard redox potential (1.23 V), which is the electrolysis voltage of water, H_2_O → H_2_ + 1/2O_2_^32^. Consequently, an electric distributed-constant equivalent circuit is established on the moist-TOCN surface, as illustrated in Fig. 4g, highlighting the intricate interactions and polarisation effects at the molecular level within the TOCN supercapacitor system.

## Conclusions

We successfully demonstrated the exceptional electric energy storage capability of moist TOCN supercapacitors, at an energy density of 8.55 J/m^2^. This high performance is primarily attributed to the formation of an electric double layer at the electrolyte interface due to electrical polarisation. The adherence of pseudo-solid water molecules to the hydrophilic cellulose surface facilitates the formation of this layer upon charging. Our study confirms the critical role of molecular interactions in boosting the energy storage efficiency of TOCN supercapacitors, thus opening up promising prospects for future energy storage technologies. Future studies will address the long-term stability of these supercapacitors and the scalability of their production process for commercial use.

## Methods

The wetting behaviour and electrochemical properties of four types of CNFs were investigated: mechanically defibrated kenaf, conifer, and bamboo, along with chemically defibrated conifer (TEMPO-oxidised cellulose nanofibre, TOCN). Each sample was moistened for 18 ks at 298 K under a humidity of 95% through a humidity-control device (Temperature and Humidity Chamber, IW223, Yamato Scientific, Tokyo). Anhydrous alcohol was dropped onto the sample as the wetting liquid. An amorphous cellulose fibre (ACF) specimen (thickness = 10 μm), covering an area of 1.8 cm^2^ with an apparent solid density of 92.67% (calculated as 1.3622/1.47 × 100), was prepared on an Al substrate through slip casting and subsequently dried in a ventilated oven at 363 K. The electrochemical behaviours, including AC impedance and DC charging/discharging properties, were assessed using a potentiostat/galvanostat (SP-150, BioLogic Science Instruments) at 10 V and 1 and 10 μA for approximately 60 s, with a charging current of 2 mA for 50 s at 293 K. The current–voltage (*I*–*V*) and resistivity–voltage (*R*–*V*) characteristics were measured under DC voltages from − 100 to 100 V at a seep rate of 1.24 V/s using a Precision Sources/Measure Unit (B2911A, Agilent).

### Supplementary Information

Supplementary Information.

## Data Availability

The datasets used and/or analysed during the current study are available from the corresponding author upon reasonable request.
