# Supplementary material for: High-energy density cellulose nanofibre supercapacitors enabled by pseudo-solid water molecules
Source: Sci Rep. 2024 May 6;14:10350. doi: 10.1038/s41598-024-61128-w (PMC11074157; doi:10.1038/s41598-024-61128-w)
Supplement: Supplementary file 1 — Supplementary Information. [file 41598_2024_61128_MOESM1_ESM.pdf]

Supplementary Information

High-energy density cellulose nanofibre supercapacitors enabled by pseudo-solid water molecules

Mikio Fukuhara [1 *], Tomonori Yokotsuka[1], Masahiro Morita[2], Tatsunori Ito[2], Minoru Yada[2], Takeshi Nakatani[2], Toshiyuki Hashida[1]

1.*New Industry Creation Hatchery Center, Tohoku University, Aoba, Sendai 980-8579, Japan*

2. *Fuji Innovative Materials Research Laboratory, Nippon Paper Industries, Co. Ltd., Fuji417-8520, Japan*

S1. Methods

The CNF specimens of mechanically defibrated conifer, bamboo, and kenaf were fabricated on a Si substrate by spin coating, which were performed at a speed of 400 rpm for 5 s, using a 2 % (w/v) CNF (20-60 nm in diameter) /water dispersion (conifer:13A21-nanoforest-S-1NBC, bamboo:10E21-nanoforest-5-1BBRB, Chuetsu Pulp Industry, Japan) and 1 % (w/v) CNF/water dispersion for kenaf. After kenaf chips were grinded by a wet mill, the sediment was miniaturised through thin-film swirling high-speed milling at 3,000 rpm. The average diameter is approximately 20 nm[33]. The CNF films were dried in a ventilated oven at 363 K. The thickness of the sample was then measured at three points using a micrometer, and the average value was used. The transparent conifer CNF was obtained by the TEMPO oxidation method of Nippon Paper Industries[12]. The specimens of three kinds of mechanically defibrated and TEMPO oxidised CNFs comprise amorphous cellulose phases. The 12–mm wide, 10 μm–thick, and 15 mm–long specimens were mechanically sandwiched between two Al electrodes.

Nitrogen adsorption was measured at increasing pressures, and adsorption-desorption isotherms were plotted against relative pressure (P/P_0, where P is equilibrium pressure and P_0 is nitrogen's saturation pressure). The cell, once degassed, was affixed to the device, cooled to liquid nitrogen temperature (77K), and subjected to controlled pressures of nitrogen gas. Pore size distribution analysis was conducted with BELMaster software, employing "Smoothing" for moving average, "No-assumption" on distribution function, "Solid and Fluid Def. Pore Size" for size definition, and "Slit-C-Adsorption" kernel via GCMC (Grand Canonical Monte Carlo method).

S 2 . Evaluation of surfaces for conifer, bamboo, kenaf and TOCN

Since the specific surface area of the conifer and bamboo sheet surfaces could not be measured using the BET adsorption method, the pore distribution was measured using a method. The results are shown in Fig. S1. The pore distribution on the sheet surface of

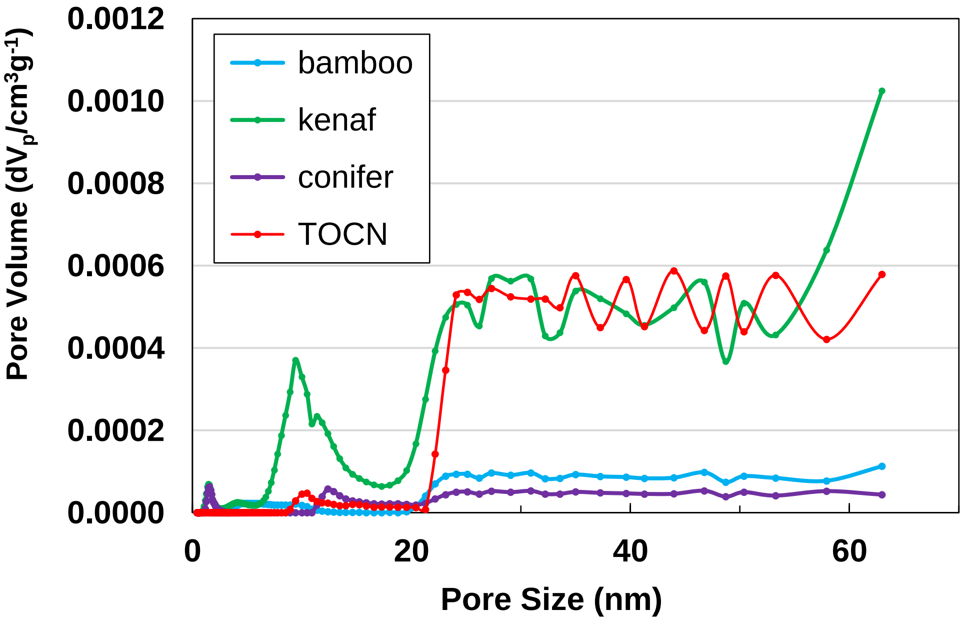

Fig. S1 Pore size distribution on surfaces of bamboo, conifer, kenaf and TOCN.

kenaf and TOCN is about five times larger than that of conifer and bamboo. That is, the specific surface area of the former is about 5 times larger than that of the latter.

S3. Charging behaviours

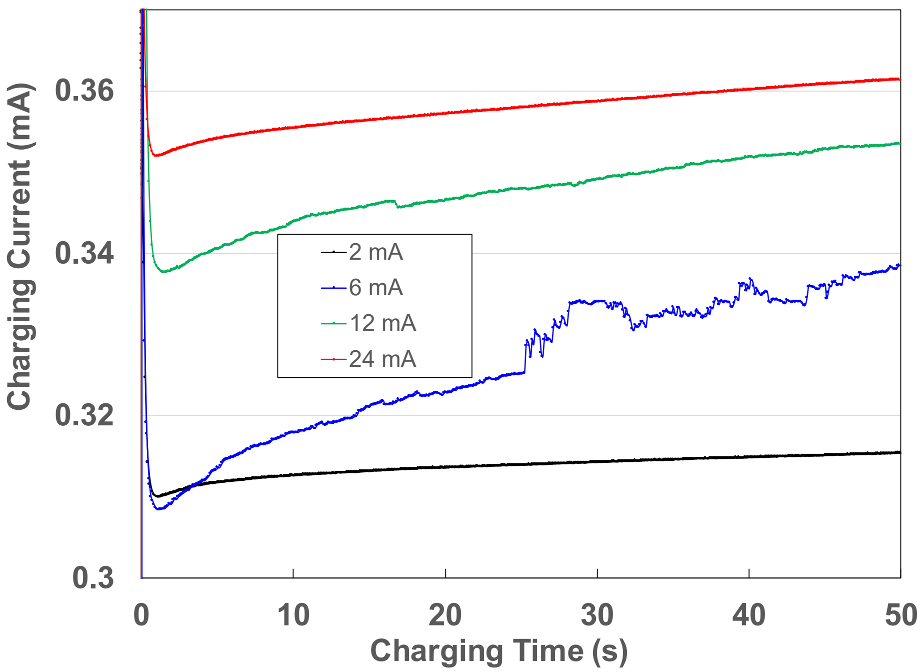

Fig. S2 Change in current passing through the sample with respect to change in charging current.

It is meaningful to investigate the charging characteristics of this new energy storage device, which has an electric double layer of bound water. Under a constant voltage of 10 V, the charging current was changed to 2, 6, 12, and 24 mA, and changes in the current flowing through the sample for 50 seconds were measured. The results are shown in Fig. S2. The current values increase as the charging current increases, and at 24 mA it almost saturates after 50 seconds. As is clear from discharging curves for moist TOCN specimens after 24 mA-10, 20, and 30 V charging in Fig. 2b, in order to further improve the energy density, it is desirable to charge with a current of 24 mA or more and a voltage of 30 V or

more. For this purpose, it is necessary to further increase the withstand voltage of the sample.

S4. Cellulose structure that prevents water and organic solvents from penetrating

Cellulose is a chair-typed polymer in which six-membered D-glucose (glucose) rings are linked in a straight chain through single bonds between carbons. The equatorial side facing the OH group is hydrophilic, and the axial side facing the hydrogen atom is hydrophobic. When the cellulose is assembled, stable aggregates are created in which molecules are bonded together by hydrogen bond interactions between hydrophilic surfaces and van der Waals interactions between hydrophobic surfaces. Since the molecules are tightly packed together without any gaps, the structure makes it difficult for water and organic solvents to enter. Therefore, although cellulose itself is a hydrophilic polymer, it has the unique property of having low solubility in various liquids.

From the above considerations, even if the vapor invades the CNF film, it cannot penetrate into the CNF itself, and is localized at the interface between the metal electrode and the CNF.

S5. Evaluation of electrostatic contribution

To estimate the electrostatic contribution of the moist TOCN supercapacitor, the relaxation times (time constants) of the three semicircles of the Nyquist diagram in Fig.

3c were determined. The relaxation time of each semicircle was calculated from the frequency f_{max} of the semicircle apex using $RC_{total} = 1/2\pi f_{max}$. The results are shown in Table S1.

Table S1 Frequencies f_{max} (Hz), relaxation time (s), and kinds of polarization for Nyquist diagram of Fig. 3c.

	Frequency f_{max} (Hz)	Relaxation time (s)	Kind of polarization
1st large semicircle	20,039	7.96×10^{-6}	orientational
2nd middle semicircle	271.5	5.85×10^{-4}	interfacial
3rd small semicircle	0.353	0.45	space charge

The large first peak is considered to be due to orientation, because the TOCN sample used in this study is a fiber with an aspect ratio of ~100. From the frequency of 271.4 Hz in the second middle semicircle, it seems to be due to interfacial polarization due to electrons passing through the interface between CNFs in the audio frequency range[34]. From the third small semicircle, it is thought that this is due to space charge polarization between the electrode and the electric double layer of pseudo-solid water.

S6. Coulomb efficiency

Information on the Coulombic efficiency must be provided. Table 2 shows the ratio of the discharge amount when discharging at a constant current of 24 mA to the charge amount when charged at 10 V - 2 mA for 50 seconds for devices made using TOCN, conifer, bamboo, and kenaf.

Table S2 Coulombic efficiency

specimens	TOCN	conifer	bamboo	kenaf
Coulombic efficiency (%)	153.8	13.0	25.7	124.4

The large value of TOCN may be due to the effect of bound water bonded to COONa groups that bridged between nanofibrils[35]. On the other hand, the lower values for samples made from conifer and bamboo are likely due to leakage of electrical storage from the samples.

S7. Electrocapillarity effect

We consider a reason why a huge electric charge can be stored between CNF aggregate and the electrode, using an electrocapillary equation[36]. Electrocapillary forces are related to the change in interfacial tension that occurs when a voltage is applied across the interface of two matters. Capacitance C is given by $C = \varepsilon A/d$, where ε is the dielectric constant, A the surface area of the electrode, and d the distance between a pair of electrodes[37]. To increase the storage capacity, it is very effective to decrease the distance in nanometer technology. Since the potential difference at the metal surface depends on

the external charge derived from electrocapillary forces, the charge density (specific capacitance σ) can be defined by $\sigma = -\frac{\partial \gamma}{\partial E}$, where γ is interfacial tension and E is potential.

Since $C = \frac{\partial \gamma}{\partial E}$, we get

$$\sigma = \int C \, \mathrm{d}E = \int \frac{\varepsilon A}{d} = - \frac{\varepsilon AE}{d} \tag{1}$$

Dielectric constant ε of the sample is 4.38 F/m (0.45 s/8,560 Ω/1.2 × 10^{-5} m) and electrode area 1.8×10^{-4} m^2. Hence, the electric charge density for E =1 V is

$$\sigma = - (4.83 \times 1.8 \times 10^{-4} \times 1)/d = - 8.017 \times 10^{-4}/ d. \tag{2}$$

The specific capacitance increases parabolic with decreasing distance between the

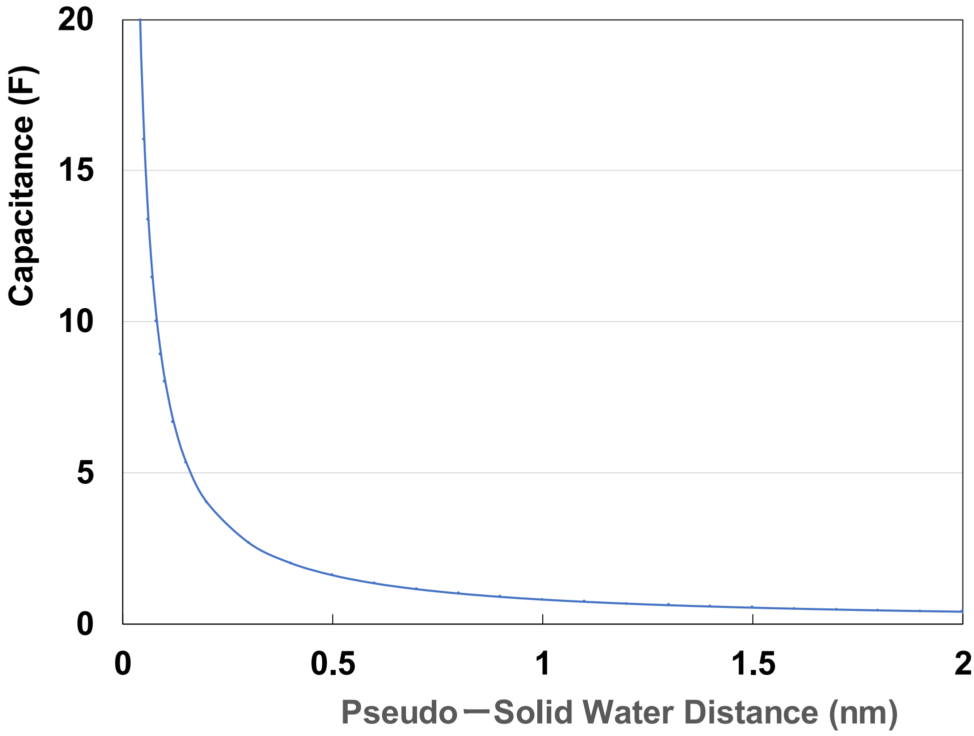

Fig. S3 Relationship between the stored capacitance and the distance between the electrode and water assuming the presence of pseudo-solid water between the CNF aggregate and the electrode

electrode and water assuming the presence of pseudo-solid water between the CNF aggregate and the electrode (Fig.S3). When electrical charges are physically stored in solid water, it is easy to imagine that the electrical charges can be stored as a polarized solid in addition to external charging. It is known as electroadsorption[38]. If the thickness of the pseudo-solid aqueous phase is 0.86 [26] and 0.31 nm [27], the specific capacitance is calculated to be 0.932 and 2.578 MF, respectively.

S8. *I-V* Characteristics of moist conifer specimen.

The *I-V* characteristics of the moist conifer specimen relative to the moist TOCN sample in Figure 2d are shown in Fig. S4. In the negative voltage range from -50 V to 0 V, almost no

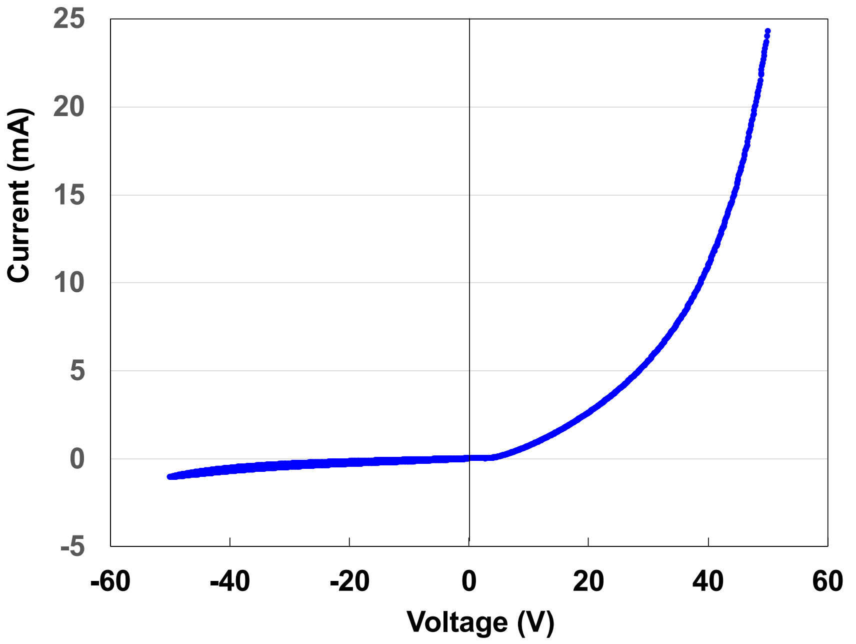

Fig. S4 *I-V* characteristic curve between -50 to + 50 V for the moist conifer specimen.

current flows, but in the positive voltage range from 0 V to +50 V, it exhibits a rectifying effect in which the current increases.

References

33. Fukuhara, M. *et al*. A novel n-type semiconducting biomaterial, *Sci. Rep.*, **12**, 21899 (2022). doi: 10.1038/s41598-022-26582-4

34. Muto, T., Sugihara, M., Goto, T. & Machi, Y. *Electronic Materials Devices*, p. 161 (Ohom, Tokyo, 1986).

35. Fukuhara, M. *et al*. High-energy storage capacity of cellulose nanofiber supercapacitors using bound water, *Sci. Rep*., **13**, 16600 (2023). doi: 10.1038/s41598-023-43222-7

36. Grahame, B. C. The electrical double layer and the theory of electrocapillarity, *Chem. Rev*. **41**,441−501 (1947).

37. Conway, B. E., Electrochemical Supercapacitors: Scientific, Fundamentals, and Technological Applications (Kluwer, New York, 1999). dx.doi.org/10.1007/978-1-4757-3058-6

38. Dupeyrat, M. & Nakache, E., Electrocapillarity and electroadsorption, *J. Colloid Interface Sci*. **73**, 332−344 (1980). doi.org/10.1016/0021-9797(80)90080-6
